# KDM4B histone demethylase and G9a regulate expression of vascular adhesion proteins in cerebral microvessels

**DOI:** 10.1038/srep45005

**Published:** 2017-03-22

**Authors:** Ji-Young Choi, Sang-Sun Yoon, Sang-Eun Kim, Sangmee Ahn Jo

**Affiliations:** 1 Department of Nanobiomedical Science & BK21 PLUS NBM Global Research Center for Regenerative Medicine, Dankook University, Cheonan 330-714, South Korea; 2 Department of Pharmacology, College of Pharmacy, Dankook University, Cheonan 330-714, South Korea

## Abstract

Intercellular adhesion molecule 1 (ICAM1) mediates the adhesion and transmigration of leukocytes across the endothelium, promoting inflammation. We investigated the epigenetic mechanism regulating ICAM1 expression. The pro-inflammatory cytokine TNF-α dramatically increased ICAM1 mRNA and protein levels in human brain microvascular endothelial cells and mouse brain microvessels. Chromatin immunoprecipitation revealed that TNF-α reduced methylation of histone H3 at lysines 9 and 27 (H3K9 and H3K27), well-known residues involved in gene suppression. Inhibition of G9a and EZH2, histone methyltransferases responsible for methylation at H3K9 and H3K27, respectively as well as G9a overexpression demonstrated the involvement of G9a in TNF-α-induced ICAM1 expression and leukocyte adhesion and transmigration. A specific role for KDM4B, a histone demethylase targeting H3K9me2, in TNF-α-induced ICAM1 upregulation was validated with siRNA. Moreover, treating mice with a KDM4 inhibitor ML324 blocked TNF-α-mediated neutrophil adhesion. Similarly, TNF-α-induced VCAM1 expression was suppressed by G9a overexpression and KDM4B knockdown. Collectively, we demonstrated that modification of H3K9me2 by G9a and KDM4B regulates expression of vascular adhesion molecules, and that depletion of these proteins or KDM4B reduces inflammation-induced leukocyte extravasation. Thus, blocking ICAM1 or KDM4B could offer a novel therapeutic opportunity treating brain diseases.

The blood-brain barrier (BBB) is present in all capillaries of the central nervous system (CNS) parenchyma. It is composed of microvascular endothelial cells connected by tight junctions, and restricts the passage of certain molecules to the CNS. In contrast to endothelial cells from other organs, junctional complexes of brain endothelial cells consist of tight junctions and adherens junctions, which provide the barrier function^1^. Claudins (1, 3, 5, and 12) and occludins are integral membrane proteins found at tight junctions that interact with the actin cytoskeleton through scaffolding proteins such as zonula occludens-1. Adherens junction proteins, such as VE-cadherin, mediate cell-to-cell contacts required to maintain barrier properties, and to regulate the expression of tight junction genes in brain endothelial cells. Reduction in VE-cadherin expression resulting in BBB disruption at post-capillary venules occurs in neurodegenerative disorders, such as Alzheimer’s disease (AD), Parkinson’s disease (PD), amyotrophic lateral sclerosis, and multiple sclerosis^2^,^3^,^4^,^5^.

Leukocyte transmigration across the endothelium is a key event in the pathogenesis of inflammatory diseases such as atherosclerosis, ischemic stroke, multiple sclerosis and experimental autoimmune encephalomyelitis^6^,^7^,^8^. Crosstalk between immune cells and endothelium is an essential element in mediating leukocyte infiltration during both normal immune surveillance and under pathological conditions. However, the potential importance of leukocyte traffic in these diseases is not fully understood, but induction of adhesion molecules such as intercellular adhesion molecule 1 (ICAM1) and vascular cell adhesion protein 1 (VCAM1) by inflammatory cytokines contributes to leukocyte adhesion by binding to leukocyte integrins, lymphocyte function-associated antigen-1 (LFA-1), macrophage antigen-1 (MAC-1), and very late antigen-4 (VLA-4), thus facilitating the transmigration of leukocytes across vessels. The relative participation of these adhesion molecules in transmigration likely determines the extent of recruitment for different leukocytes subpopulations. ICAM1 was identified as a key adhesion protein in the recruitment of lymphocytes to the CNS^9^.

The involvement of adhesion molecule induction in pathogenesis of neurodegenerative brain disease is likely avid. Upregulation of ICAM1 and lymphocyte infiltration into brain are features of in PD^10^,^11^. In case for AD, soluble ICAM1 in the plasma of AD patients is higher than that in mild cognitive impairment subjects^12^. In addition, the control of neutrophil binding to brain microvessels by blocking integrin activity retards AD pathogenesis and reverses memory loss^13^. Since ICAM1 increases the activity of matrix metalloproteinases, while reducing the amount of VE-cadherin^14^,^15^, these results suggest that repression of ICAM1 is required to maintain a sufficient level of VE-cadherin to inhibit leukocyte transmigration across the endothelium. With regard to this it is reported that VE-cadherin phosphorylation at tyrosine 685 or 731 by ICAM1 mediated-Src/Pyk2 signaling induces the passage of blood cells across endothelial cells^16^,^17^. Thus, molecular mechanisms of ICAM1 in controlling leukocyte adhesion could be a key therapeutic target for neurodegenerative diseases.

Histone methyltransferase, which transfers 1–3 methyl groups from S-adenosyl methionine to lysine or arginine residues of H3 and H4 histones, has been related to both transcriptionally active and inactive chromatin, depending on the methylated residue position and its degree of methylation (mono-, di-, or tri-methylation). Lysine residues 4, 9, 27, and 36 are major sites of H3 methylation, whereas lysine 20 is the site of methylation on H4. Tri-methylation of lysine 4 on histone H3 (H3K4me3) is associated with transcriptional activity, whereas di-and tri-methylation of H3K9 (H3K9me2 and H3K9me3) and tri-methylation of H3K27 (H3K27me3) are associated with transcriptional inactivity. Several studies have reported that reducing H3K9me2 or H3K27me3 in conditions of gene silencing reactivates gene expression^18^,^19^,^20^,^21^.

In the present study, we investigated the epigenetic regulatory mechanism of how inflammation regulates ICAM1 expression in endothelial cells and the role of ICAM1 in the pathogenesis of brain diseases such as AD. We demonstrated that a reduction in H3K9me2 and H3K27me3 mediates TNF-α-induced ICAM1 expression, and that histone demethylase KDM4B and histone methyltransferase G9a are the enzymes responsible for this regulation. Increased leukocyte adhesion/transmigration across the endothelium by TNF-α was blocked by KDM4Bknock down or KDM4inhibition, suggesting a pivotal role for H3K9me2 in ICAM1 expression during TNF-α-mediated vascular inflammation.

## Results

### ICAM1 expression is enhanced by TNF-α in both brain microendothelial cells and microvessels

**Figure 1:**
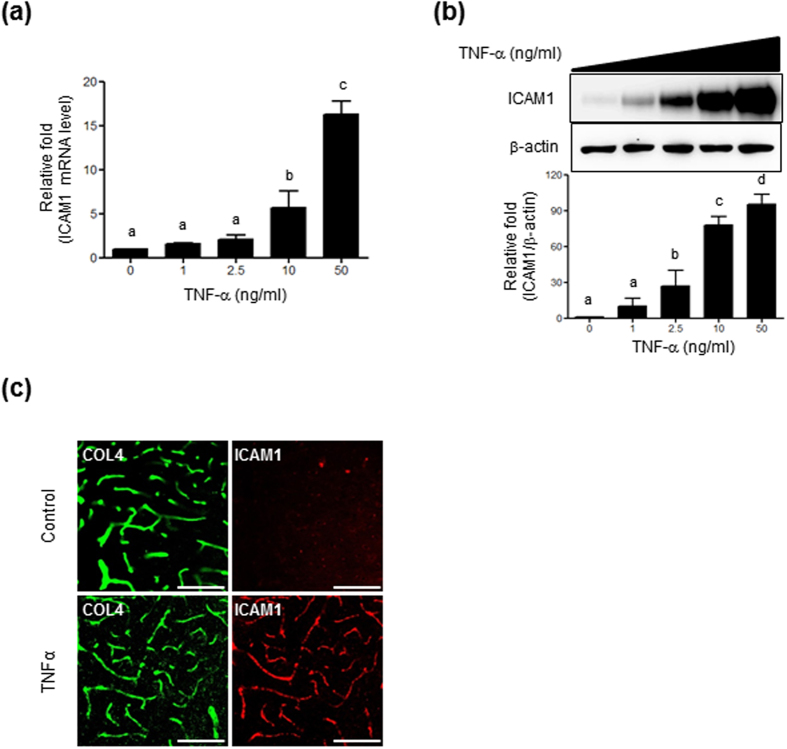
Effect of TNF-α on ICAM1 expression. HBMVECs were seeded in a 6-well plate and treated with various doses of TNF-α for 24 h. (**a**) ICAM1 mRNA levels. Levels were normalized to GAPDH and expressed relative to ICAM1 from non-treated controls (n = 3). (**b**) ICAM1 protein levels determined by western blot analysis using 20 μg of cell lysates. Quantification was performed using densitometry (Image J software). Results were normalized to β-actin. Uncropped blots are found in Supplementary Fig. S5. (**c**) Sections from saline or TNF-α -injected mouse brains immunostained with vessel markers; anti-collagen type IV (COL4; green) and anti-ICAM1 (red) antibodies. Scale bars, 100 μm. Bars represent mean ± SEM (n = 3). Data were analyzed by a one-way ANOVA followed by a *post hoc* Student-Newman-Keuls test. The different characters denote significant differences (p < 0.05) among the groups.

First, we examined whether TNF-α upregulates ICAM1 expression in microendothelial cells. Treatment of HBMVECs with TNF-α (1, 2.5, 10, or 50 ng/mL, for 24 h) upregulated ICAM1 mRNA expression in a dose-dependent manner, resulting in 5.7- and 16.2-fold increases at 10 ng/mL and 50 ng/mL, respectively (Fig. 1a). TNF-α treatment at 2.5 ng/mL also induced a significant increase in ICAM1 protein, but a maximal increase was observed at 50 ng/mL (Fig. 1b). Based on these results, the effective dose 50 (ED_50_) for TNF-α to induce ICAM1 expression was estimated as 10 ng/mL. Thus, all subsequent experiments were performed at this concentration. We then examined whether TNF-α upregulates ICAM1 expression in mouse brain microvessels by immunohistochemistry. Eight week-old C57BL/6 mice were injected with TNF-α (9 μg/kg). After 24 h, immunofluorescence staining for ICAM1 and collagen type IV as a vessel marker were performed. ICAM1 immunoreactivities were more prominent in vessels from TNF-α-injected mice than in saline-treated control animals (Fig. 1c).

### Histone methylation is associated with TNF-α-induced ICAM1 expression

**Figure 2:**
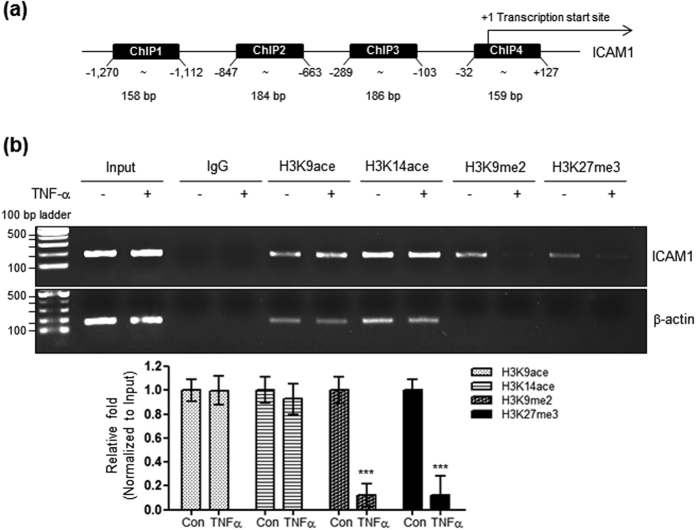
Effect of TNF-α on histone H3 lysine acetylation or methylation within the *Icam1* promoter in HBMVECs. (**a**) Primer sites (−1,270 to −1,112, 158 bp; −847 to −663, 184 bp; −289 to −103, 186 bp; −32 to +127, 159 bp) for ChIP are numbered relative to the main transcription start site (+1). (**b**) Genomic DNA was isolated from HBMVECs treated with 10 ng/mL of TNF-α for 24 h. A ChIP assay was performed with antibodies specific for H3K9ace, H3K14ace, H3K9me2, or H3K27me3 (listed in Table 3). The DNA isolated after ChIP was used for a semi-quantitative PCR with ChIP 2 primers (listed in Table 2). Input represents amplification of the total input DNA from whole cell lysates. The amount of DNA after ChIP was normalized to the input DNA level. Quantification was performed using densitometry (Image J software). Results were normalized to β-actin. Bars represent mean ± SEM (n = 3). ^***^Denotes statistically significant difference at *p* < 0.001. Uncropped blots are found in Supplementary Fig. S6.

It was reported that ICAM1 expression was affected by trichostatin A, a histone deacetylase (HDAC) inhibitor, in tumor-conditioned endothelial cells^22^, suggesting its regulation by histone acetylation. Thus, a ChIP assay was performed with specific primer sets recognizing the human *Icam1* promoter. A previous study on human umbilical vein endothelial cells showed that regions −941 to −574 and −277 to −105 in the *Icam1* 5′-flanking region might contain important elements regulating TNF-α-dependent ICAM1 gene expression^23^. To identify the regions which plays an important role in TNF-α-mediated histone modifications and therefore ICAM1 gene expression, we designed four primer sets (ChIP1, −1,270 to −1,112; ChIP2, −847 to −663; ChIP3, −289 to −103; ChIP4, −32 to +127) within the −1.2 kb region of the *Icam1* 5′ flanking region as shown in Fig. 2a; primer sites are numbered relative to the main transcription start site (+1). Results obtained using ChIP2 showed that TNF-α did not affect acetylation levels at lysines 9 and 14 of histone H3 (H3K9ace and H3K14ace), known as the activation histone code within the *Icam1* promoter region. However, it significantly reduced the levels of H3K9me2, H3K9me3, and H3K27me3, known as the suppression histone code (Fig. 2b; Supplementary Fig. S1). Results obtained using ChIP primers 1, 3, and 4 were similar to those of ChIP2 (Supplementary Fig. S2), except that H3K27me3 levels were unaltered by TNF-α (Supplementary Fig. S2), when using ChIP4 primers. A previous study showed an important role for the −941 to −574 and −277 to −105 regions of the *Icam1* promoter for activity. A significant role for transcription factor heat shock factor 1 (HSF1)^24^, which has binding sites that are rich within the ChIP2 primer region (−847 to −663), was also demonstrated. Thus, all subsequent experiments were performed with ChIP2 primers. Taken together, these results suggest that histone methylation, such as H3K9me2 or H3K27me3, is implicated in TNF-α-induced ICAM1 expression.

### G9a is involved in the epigenetic regulation of ICAM1

**Figure 3:**
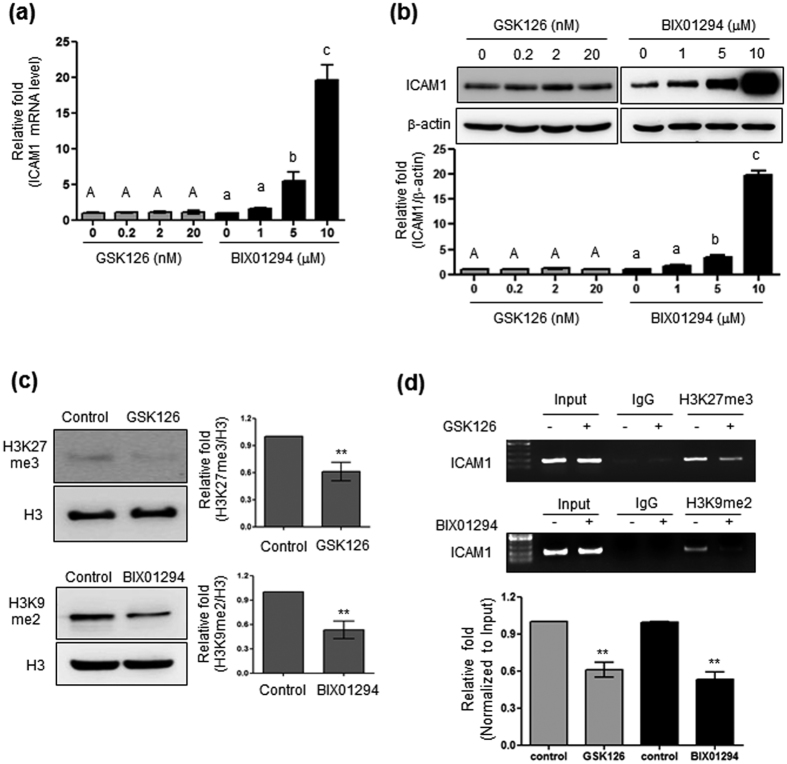
Effect of EZH2 or G9a specific inhibitors on ICAM1 gene expression. (**a**) ICAM1 mRNA levels in HBMVECs treated with GSK (0, 0.2, 2, and 20 nM) or BIX01294 (0, 1, 5, and 10 μM). Results were normalized to GAPDH and expressed relative to ICAM1 from non-treated controls. (**b**) ICAM1 protein from total cell lysates of HBMVECs treated as above. Quantification was performed using densitometry (Image J software). Results were normalized to β-actin. (**c**) H3K27me3 and H3K9me2 levels determined from 30 μg of the cell lysates (described as above) and separated by 15% SDS-PAGE. Results were normalized to histone H3. Uncropped blots are found in Supplementary Fig. S7. (**d**) Binding of H3K27me3 or H3K9me2 to the *Icam1* promoter site quantified by ChIP with anti-H3K27me3 or anti-H3K9me2 specific antibodies. Input represents the total input DNA from whole cell lysates. Bars represent mean ± SEM (n = 3). The different characters denote significant differences (p < 0.05) between the groups. ^**^Denotes statistically significant difference at p < 0.01. Uncropped blots are found in Supplementary Fig. S7.

To evaluate whether alterations of the histone code, such as H3K9me2 or H3K27me3, affect ICAM1 expression, cells were treated with GSK126 and BIX01294, which are specific inhibitors of EZH2 and G9a, methyltransferases responsible for H3K27me3 and H3K9me2, respectively. BIX01294 inhibits both G9a and GLP which is responsible for H3K9me, but has lower binding affinity to GLP responsible for H3K9me^25^. As shown in Fig. 3a, treatment with GSK126 (0.2~20 nM; IC_50_ = 9.9 nM) did not affect ICAM1 mRNA levels, whereas BIX01294 (1~10 μM; IC_50_ = 1.7 μM) mediated a dose-dependent increase in ICAM1 mRNA. Western blotting analysis also revealed the involvement of G9a, through BIX01294 treatment, in ICAM1 gene regulation; again, GSK did not result in changes in ICAM1 expression (Fig. 3b). As expected, inhibition of EZH2 and G9a activity reduced overall H3K27me3 and H3K9me2 levels, respectively (Fig. 3c), which are associated with the *Icam1* promoter (Fig. 3d). These results indicate that H3K9me2 mediated by G9a is associated with ICAM1 transcriptional regulation.

**Figure 4:**
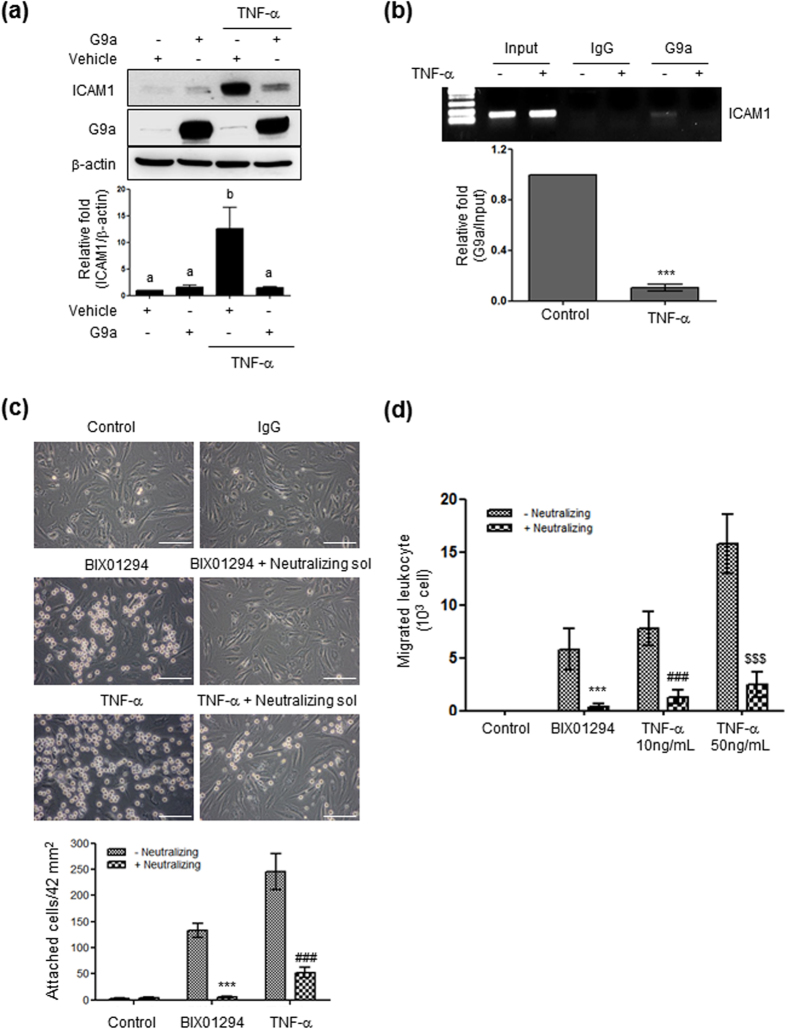
The role of G9a in the expression of ICAM1 and leukocyte adhesion. (**a**) ICAM1 protein levels in G9a-overexpressing cells treated with TNF-α for 24 h. Uncropped blots are found in Supplementary Fig. S8. (**b**) A ChIP assay performed with a G9a-specific antibody. (**c**,**d**) Photographs showing HL-60 cell adhesion and migration. HBMVECs seeded in a 12-well plate were incubated with 10 μM BIX01294 or 10 ng/mL TNF-α for 24 h, and treated with 2 μg of rabbit IgG or neutralizing solution (anti-ICAM1 rabbit polyclonal and anti-VCAM1 rabbit monoclonal) for 0.5 h before the addition of 10^5^ HL-60 cells. Uncropped blots are found in Supplementary Fig. S8. (**c**). Attached HL-60 cells were observed and counted under an inverted microscope with 400× magnification (mean ± SEM; n = 5). Scale bars, 50 μm. Values are considered significant at ^***^p < 0.001 *vs* BIX01294; ^###^p < 0.001 *vs* 10 ng/mL TNF-α. (**d**) Quantitation of HL-60 cell transmigration assay. The bar graph indicates the number of migrating HL-60 cells (mean ± SEM; n = 3–4). Values are considered significant at ^***^p < 0.001 *vs* BIX01294; ^###^p < 0.001 *vs* 10 ng/mL TNF-α; ^$$$^p < 0.001 *vs* 50 ng/mL TNF-α.

Next, we examined the role of G9a in TNF-α-induced ICAM1 expression through overexpression studies. Cells were transfected with G9a before TNF-α treatment, and ICAM1 protein levels were measured. As shown in Fig. 4a, TNF-α-induced ICAM1 expression was blocked by G9a overexpression. A ChIP assay (Fig. 4b) revealed that G9a interacted with the *Icam1* promoter region in the control conditions, but dissociated from the *Icam1* promoter upon TNF-α treatment. These results indicated that methylation of H3K9 (H3K9me2) by G9a is an important regulatory step for ICAM1 expression in HBMVECs. ICAM1 and VCAM1 are known to mediate the binding of leukocytes to endothelial cells, consequently leading to leukocyte transmigration^26^. Thus, we tested the functional consequences of TNF-α- and BIX01294-induced ICAM1 upregulation by measuring leukocyte adhesion and transmigration across HBMVECs. As shown in Fig. 4c, both TNF-α (10 ng/mL) and BIX01294 (10 μM) treatment increased HL-60 leukocyte adhesion to HBMVECs, which was blocked by neutralizing antibodies targeting both ICAM1 and VCAM1. Similarly, TNF-α (10 and 50 ng/mL) or BIX01294 treatment increased leukocyte transmigration across HBMVECs, and this increase was blocked by neutralizing antibodies (Fig. 4d).

### KDM4B is involved in epigenetic regulation of ICAM1

**Figure 5:**
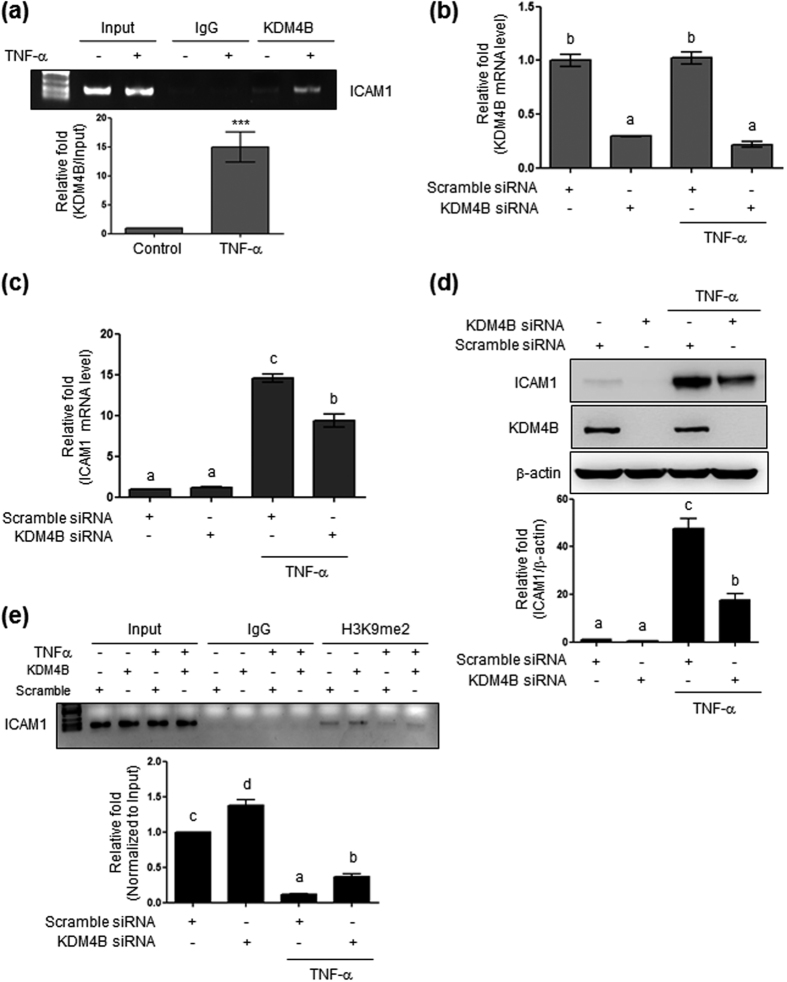
Effect of KDM4B knockdown on ICAM1 expression. (**a**) Isolation of Genomic DNA from HBMVECs transfected with 100 nM scrambled or 100 nM KDM4B siRNA followed by TNF-α treatment (10 ng/mL, 24 h). ^***^Denotes statistically significant difference at *p* < 0.001. (**b**) Confirmation of transfection efficiency through reduced KDM4B mRNA levels. (**c**) ICAM1 mRNA expression in cells transfected with scrambled or KDM4B siRNA followed by TNF-α treatment. (**d**) ICAM1 protein levels determined by western blotting using 15 μg of cell lysate and 8% SDS-PAGE. (**e**) H3K9me2 binding to the *Icam1* promoter region determined by ChIP. Results show the mean ± SEM (n = 3–4). The different characters denote significant differences (p < 0.05) among the groups. Uncropped blots are found in Supplementary Fig. S9.

In order to identify the subtypes of histone demethylases involved in TNFα-mediated hypomethylation on H3K9me2, we screened by the western blotting histone demethylases which were changed by TNF-α treatment. From the initial two experiments we found that TNF-α increased the KDM4B protein level, which led us to test the possible role of KDM4B in ICAM1 gene expression. Thus, we performed a ChIP assay and found an increased binding of KDM4B on ICAM1 promoter region after TNF-α treatment (Fig. 5a). However, quantification with additional experiments showed that both KDM4B protein and mRNA levels were not significantly altered by TNF-α (data not shown), suggesting that TNF-α did not affect KDM4B expression but recruit a binding of KDM4B to the ICAM1 promoter. We then further tested the role of KDM4B in TNF-α-induced ICAM1 expression. HBMVECs were transfected with specific KDM4B siRNA and then treated with TNF-α for 6 or 24 h. Transfection resulted in 70% reduction of KDM4B mRNA level; however, its expression was not affected by TNF-α treatment (Fig. 5b). KDM4B knockdown blocked TNF-α-induced ICAM1 mRNA and protein expression (Fig. 5c,d). The ChIP assay clearly showed that knockdown of KDM4B significantly enhanced H3K9me2 (1.42 ± 0.06) within the *Icam1* promoter region and restored TNF-α-repressed H3K9me2 (0.44 ± 0.05; Fig. 5e), when compared with control transfected with scramble siRNA. Taken together, these results suggest that the association between H3K9me2 and the *Icam1* promoter plays a pivotal role in regulating ICAM1 expression in response to TNF-α, and that G9a and KDM4B regulate this epigenetic histone modification (methylation).

### G9a and KDM4B regulate VCAM1 expression

**Figure 6:**
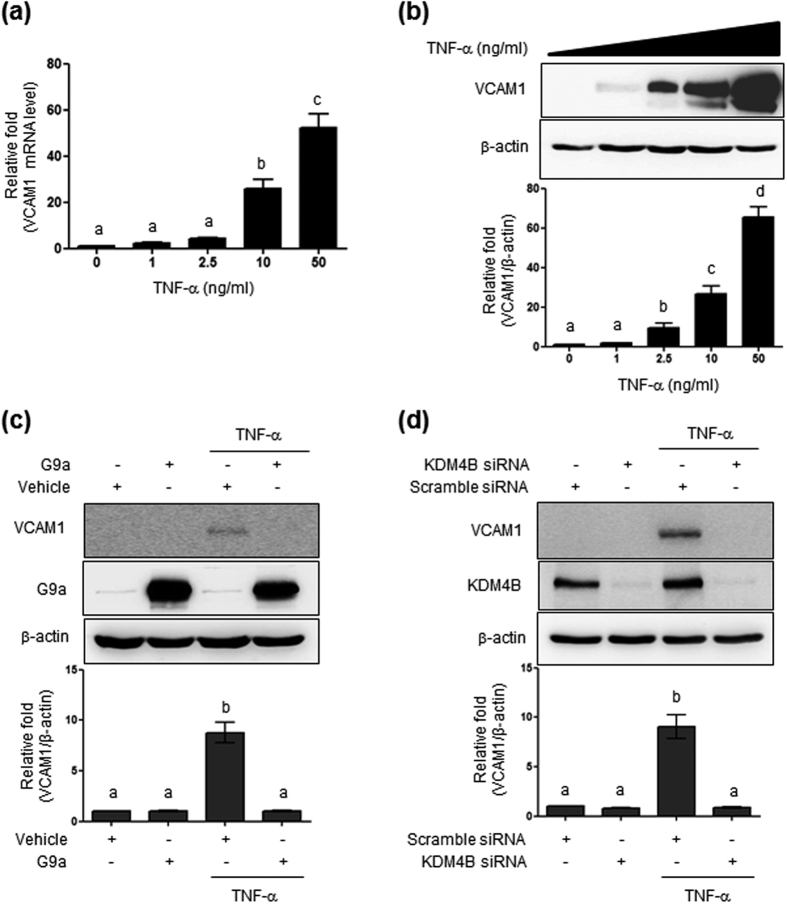
Effect of G9a overexpression and KDM4B knockdown on TNF-α-induced VCAM1 expression. (**a**) VCAM1 mRNA levels in cells exposed to diverse doses of TNF-α for 24 h. (**b**) VCAM1 protein levels determined by western blotting using 30 μg of cell lysates. (**c**) Blocking of TNF-α-induced VCAM1 expression by G9a. Efficiency of MSCV-hydro-G9a transfection was confirmed by western blotting using 20 μg of cell lysate and 8% SDS-PAGE. (**d**) VCAM1 quantification was performed through densitometry (Image J software). Results were normalized to β-actin. Bars represent mean ± SEM (n = 3). The different characters denote significant differences (p < 0.05) among the groups. Uncropped blots are found in Supplementary Fig. S10.

Others have previously observed that VCAM1 is also induced by TNF-α in endothelial cells. Thus, we examined whether TNF-α treatment affected VCAM1 expression in HBMVECs. As shown in Fig. 6a and b, TNF-α had a significant dose-dependent effect on VCAM1 mRNA and protein levels. For VCAM1, TNF-α-induced VCAM1 expression was completely suppressed by G9a overexpression and KDM4B knockdown (Fig. 6c,d).

### Inhibition of KDM4 blocks TNF-α-induced leukocyte adhesion and transmigration

**Figure 7:**
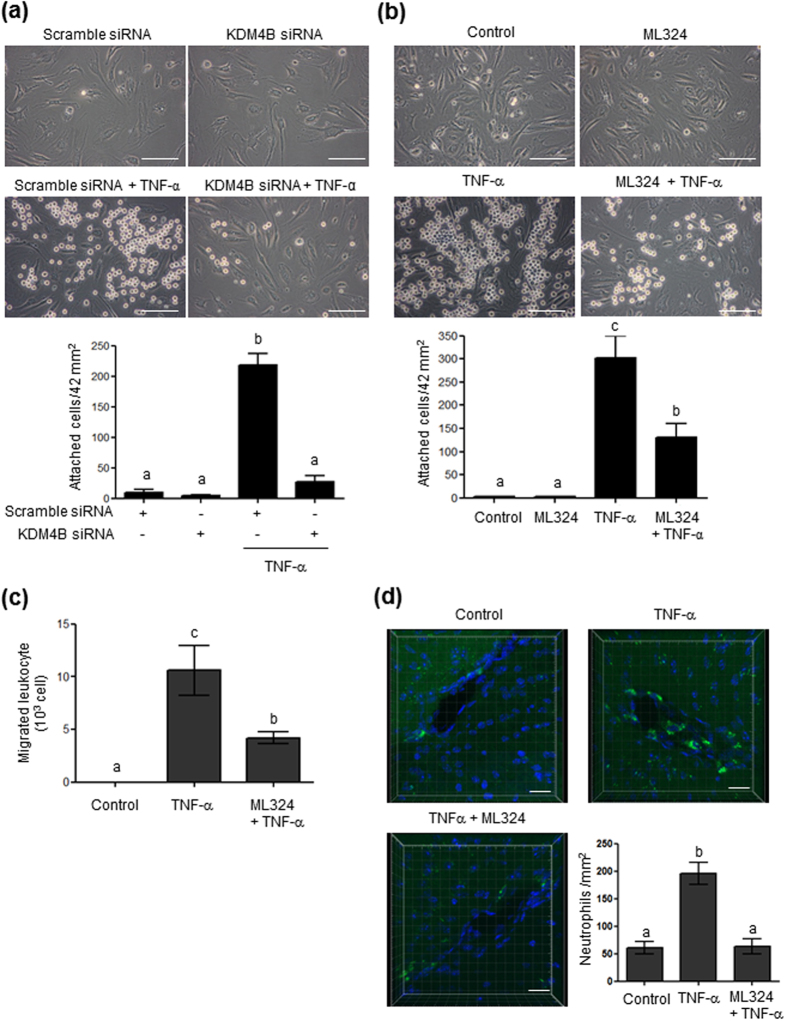
Effect of KDM4B knockdown or ML324, a pharmacological inhibitor of KDM4, on TNF-α-induced leukocyte adhesion and transmigration. (**a**) Cells transfected with 100 nM scrambled or 100 nM KDM4B siRNA were treated with Tris-HCl, pH 8.0 buffer or TNF-α for 24 h and then exposed to 10^5^ HL-60 cells at 37 °C for 1 h. (**b**) Cells pretreated with ML324 (0.2 μM) for 1 h were treated with TNF-α for 24 h and then exposed to10^5^ HL-60 cells at 37 °C for 1 h. Attached HL-60 cells were counted under an inverted microscope at 400× magnification (mean ± SEM; n = 4–5). Scale bars, 50 μm. (**c**) Quantitation of HL-60 cell transmigration assay. The bar graph indicates the number of migrating HL-60 cells (mean ± SEM; n = 3–4). (**d**) Neutrophil adherence to brain microvessels. The bar graph indicates the attached neutrophil number per mm^2^ (mean ± SEM; n = 3). Scale bars, 20 μm.

We tested the functional consequences of TNF-α-induced ICAM1 expression by measuring leukocyte adhesion and transmigration across HBMVECs, and determined whether these processes were regulated by KDM4B. As shown in Fig. 7a, TNF-α (10 ng/mL) treatment for 24 h increased leukocyte adhesion to HBMVECs, which was blocked by KDM4B knockdown. Under our experimental conditions, the transfection reagent induced cell toxicity and abolished tight junctions between endothelial cells, making it difficult to investigate the effect of KDM4B siRNA knockdown on leukocyte transmigration. Instead, we used a commercially available inhibitor of KDM4, ML324^27^. Pretreatment with ML324 partially reduced TNF-α-induced ICAM1 protein expression (Supplementary Fig. S3), and decreased leukocyte adhesion (Fig. 7b) and transmigration (Fig. 7c). These results support the involvement of KDM4B in ICAM1 regulation and leukocyte transmigration.

We also tested whether TNF-α-induced ICAM1 leads to the adhesion of leukocytes such as neutrophils to microvessels in the brain and that this adhesion is regulated by KDM4. As shown in Fig. 7d, neutrophil adhesion was found in vessels of TNF-α (9 μg/kg)-injected mouse brains, which was blocked by ML324 (0.2 mg/kg) treatment.

## Discussion

Vascular factors causing diseases such as diabetes, hyperinsulinemia, and arteriosclerosis constitute a risk for CNS disorders^28^, and endothelial dysfunction has been proposed to cause brain inflammation leading to neurodegenerative disorders such as AD and amyotrophic lateral sclerosis^4^. Although the induction of ICAM1 by inflammatory cytokines and cerebrovascular risk factors in various tissues is well documented, understanding of the molecular mechanisms involved in this regulation is limited. In the present study, we studied the epigenetic regulation of ICAM1 in HBMVECs. Our results demonstrate that TNF-α-induced ICAM1 expression is mediated through the reduction of H3K9 methylation in the *Icam1* promoter region. We also identified enzymes involved in H3K9me2 methylation and demethylation, which are G9a and KDM4B, respectively. We further investigated the functional role of KDM4B. Augmented ICAM1 protein was shown to stimulate leukocyte adhesion and transmigration across HBMVECs and reduced neprilysin protein levels (unpublished data). These responses were reversed by the suppression of endogenous ICAM1 via siRNA or through KDM4B siRNA and KDM4 inhibition. Taken together, our results support a critical role for H3K9 methylation in the expression of ICAM1, and suggest a mechanism through which inflammation-induced ICAM1 could contribute to the pathogenesis of AD.

Although several adhesion proteins such as ICAM1, VCAM1 and PECAM1 have been known to play a distinct role in leukocyte adhesion to brain ECs^9^, we are not sure at the moment which adhesion protein contributes more for leukocyte adhesion to ECs. In our previous study, we measured the ICAM1 and VCAM1 protein levels in response to TNF-α. ICAM1 protein level was increased as early as 2 h and peaked at 24 h after TNF-α treatment, and this increase was maintained quite high by 72 h^29^. In contrast, the VCAM1 protein level was induced by TNF-α at 4 h with maximal level at 6 h, and then quickly reduced to the basal level at 24 h. These results suggest different role of ICAM1 and VCAM1 in leukocyte adhesion. Considering the duration of effectiveness ICAM1 might play a more critical role in leukocyte adhesion and transmigration. In addition, the integrin α4β1 (VLA-4), αMβ2 (Mac-1), and α_L_β2 (LFA-1), receptors for vascular adhesion proteins expressed in leukocytes play a major role in leukocyte adhesion to endothelial cell. LFA-1 and Mac-1 are the most abundant integrin on neutrophils and monocyte, and VLA-4 is highly expressed in B cell. It was also reported that the main ligand for LFA-1 and Mac-1 on endothelial cell is ICAM1 and the ligand for VLA-4 is VCAM1^30^,^31^. In the present study, the cell type used for adhesion assay was HL-60, neutrophil-like cell and neutrophil isolated from mouse bone marrow. Thus, under our experimental condition, leukocyte (neutrophil) is likely to prefer to bind to ICAM1 rather than VCAM1. The understanding of functional role of ICAM1 and VCAM1in inflammation-mediated leukocyte infiltration requires further studies.

In the present study, we performed a ChIP assay using ChIP2 primers since several HSF1 binding sites (nGAAn or nTTCn) were found in the −847 to −663 region of the *Icam1* promoter. Various transcriptional factors are likely involved in TNF-α-induced ICAM1 expression. Ledebur *et al*. found that TNF-α-activated NF-κB binds to a specific sequence (TGGAAATTCCG) located between −277 and −105 of the *Icam1* promoter region and that deletion of the −941 to −574 region reduced TNF-α-induced *Icam1* promoter activity^23^. In contrast, recent reports have shown that activator protein 1 regulates ICAM1 induction in response to TNF-α in human retinal pigment epithelial cells^32^. The chromatin structure of the promoter region of inflammatory genes, such as *Icam1* and *IL-6*, is partially opened through HSF1 binding in mouse embryonic fibroblast cells^24^, suggesting a critical role for the occupancy of transcription factors at these *Icam1* loci for *Icam1* gene expression. In summary, methylated H3K9 sites are likely to be enriched within the −1.2 kb *Icam1* promoter regions, and H3K9 demethylation in these regions is likely a prerequisite to recruit the appropriate transcription factors to induce *ICAM1* gene transcription.

Although several reports have demonstrated the epigenetic regulation of ICAM1 by histone acetylation in response to inflammation during cancer andobesity^22^,^33^, no other form of epigenetic regulation of ICAM1 has been reported. Here, we describe for the first time how ICAM1 expression is epigenetically regulated. ICAM1 expression is enhanced by lowering H3K9me2 and H3K27me3 within the *Icam1* promoter region in HBMVECs. Moreover, using pharmacological inhibitors, we demonstrated that G9a but not EZH2 is implicated in ICAM1 expression. According to a recent ChIP-sequencing study, G9a and EZH2 might co-localize in the genome of human cells to co-regulate gene suppression^34^. G9a and G9a-like protein, which are responsible for H3K9me2/me3, have been reported to control the PRC2 complex containing EZH2 and H3K27me3^35^. Thus, alteration of H3K27me3, in response to TNF-α in HBMVECs, is likely to be mediated by G9a, although definitive proof of this will require further experiments. Nonetheless, our data clearly show that TNF-α-mediated ICAM1 induction depends on H3K9me2. Genome-wide ChIP sequencing has revealed that H3K9me2, H3K9me3, and H3K27me3 are enriched in transcriptionally silenced regions of the genome^36^. A reduction in overall H3K9me2 in *Drosophila*, caused by tau-induced heterochromatin loss, leads to activation of silenced genes^37^. Thus, gene reactivation is linked to demethylation through activated histone demethylases^38^. To date, more than 10 demethylases targeting H3K9me2, such as KDM1A-B, KDM3A-B, KDM4A-D, and KDM7A-C have been identified. From our experimental evidence, we conclude that KDM4B is implicated in TNF-α-induced epigenetic regulation of ICAM1 (Fig. 5), even though TNF-α upregulates KDM1B and KDM7A gene expression)^29^.

Recent studies have identified histone demethylases associated with cancers and have suggested that compounds inhibiting histone demethylase activity might have a therapeutic potential against cancers. Harris *et al*.^39^ showed that inhibition of KDM1A by tranylcypromine and two of its analogues impair the colony forming ability of acute myeloid leukemia cells. In another study, depletion of histone demethylase KDM4C was shown to decrease the growth of cancer cells, such as KYSE150 (human esophageal squamous cell carcinoma) and U2OS (human osteosarcoma)^40^. In the present study, we propose that KDM4B histone demethylase is a promising therapeutic target for AD. Specific modulation of H3K9 methylation by KDM4B could block ICAM1 gene expression induced by several vascular factors such as inflammation, high blood glucose levels, and hypoperfusion, which are well-known risk factors for AD.

It was reported that ICAM1 and VCAM1 levels were higher in peripheral blood of AD patients than in controls^41^,^42^, and that ICAM1 accumulation was found in the area surrounding fibrillary β-amyloid plaques in transgenic Tg2576 mouse brains^43^. These data suggested a relationship between enhanced ICAM1 and VCAM1 levels and AD pathogenesis, but no crucial evidence has been reported. A recent report showed that controlling neutrophil binding to brain microvessels, by blocking integrin activity, retarded AD pathogenesis and mitigated memory loss^13^. Muradashvili *et al*.^44^ reported that increased cerebrovascular permeability through downregulation of VE-cadherin was associated with memory loss. In addition, our present data showed a critical correlation between ICAM1 expression and neutrophil infiltration through vessels which could lead to inflammation in the brain. Thus, our data and previous reports collectively suggest that binding of integrin and ICAM1 after inflammation is an early step in the pathogenesis of AD.

In the present study we used TNF-α at 10 ng/mL which is higher than the circulating TNF-α concentration in the blood (0.05 or 0.5 ng/mL). A previous study showed that the TNF-α levels in the blood from normal subjects and AD patients are 6.8 and 10.6 pg/mL, respectively^45^. However, the blood cytokine level may reach much higher concentration at sites of release as shown from a study that lipopolysaccharide stimulates a release of TNF-α by blood cells from AD patients at concentrations of 2,436~4,034 pg/mL^45^. In addition, the dose-response study showed that ED_50_ of TNF-α was approximately 10 ng/mL. At 1 ng/mL, the lowest concentration examined, TNF-α treatment induced ICAM1 expression slightly (9.8 ± 3.9 fold) but not sufficiently (Fig. 1a,b). Similar observation was found for leukocyte adhesion under our experimental condition (Supplementary Fig. S4). Thus, we selected a slightly higher concentration, 10 ng/mL to induce enough ICAM1 protein to facilitate leukocyte adhesion on endothelial cell. This concentration was adopted in many other studies to see ICAM1 upregulation in human lung microvascular endothelial cells and human umbilical vein endothelial cells^46^,^47^.

**Figure 8:**
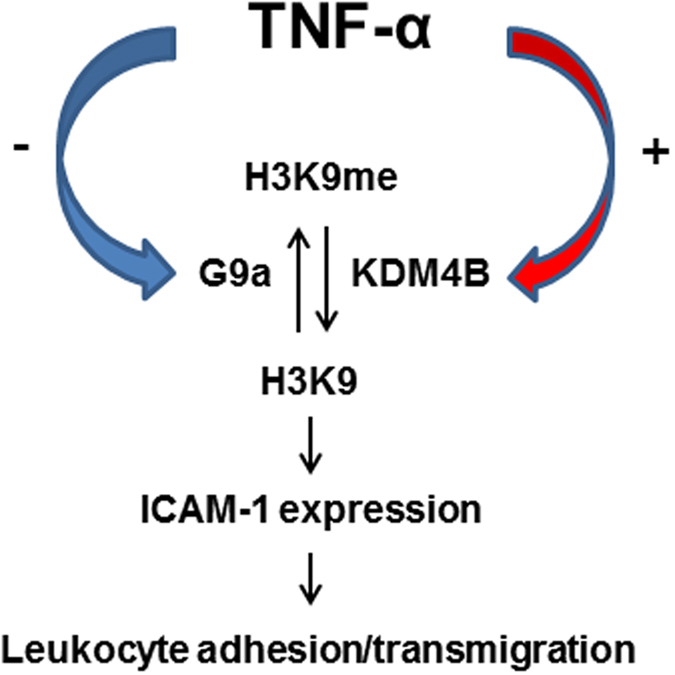
A schematic illustration of the molecular mechanism of TNF-α-induced ICAM1 expression and leukocyte adhesion/transmigration in HBMVECs. TNF-α reduces the interaction between G9a and the *Icam1* promoter region, while enhancing the interaction with KDM4B. This contributes to decreased H3K9 methylation of the *Icam1* promoter region and consequent ICAM1 expression, which lead to leukocyte adhesion/transmigration across endothelial cells.

In conclusion, our results demonstrate that TNF-α regulates ICAM1and VCAM1 expression through modification of both KDM4B and G9a activity, which results in decreased H3K9me2 levels and in turn the upregulation of ICAM1 expression (Fig. 8). Our data suggest the potential of KDM4B inhibition for brain disease such as AD by blocking ICAM1 and VCAM1-induced extravasation.

## Materials and Methods

### Reagents and plasmid

Human TNF-α, phenylmethylsulfonyl fluoride (PMSF), dimethyloxalylglycine, and triton X-100 were purchased from Sigma (St. Louis, MO). BIX01294 was obtained from Axon Medchem (Groningen, The Netherlands). GSK126 and ML324 were from Cayman (Ann Arbor, MI). Normal goat serum and mounting medium were obtained from Vector Laboratories Inc. (Burlingame, CA). Alexa Fluor^®^ 594 goat-anti-mouse IgG and Alexa Fluor^®^ 488 goat-anti-rabbit IgG were purchased from Molecular probes (Eugene, OR). Anti-ICAM1 mouse monoclonal and rabbit polyclonal antibodies were from Santa Cruz Biotechnology, Inc., (Santa Cruz, CA). Anti-G9a and anti-KDM4B rabbit polyclonal antibodies were from Cell signaling (Danvers, MA). Ant-laminin rabbit polyclonal, anti-β-actin mouse monoclonal, and anti-VCAM1 rabbit monoclonal antibodies were from Abcam (Cambridge, UK). Culture media, M199 and RPMI 1640, fetal bovine serum (FBS), and antibiotics were purchased from Gibco (Carlsbad, CA). MSCVhygro-F-G9a was a gift from Kai Ge (Addgene plasmid # 41721).

### Cell culture

Human brain microvascular endothelial cells (HBMVECs) were obtained from Cell Systems (Kirkland, WA) and were grown on attachment factor-coated plates in CSC complete serum free medium (Cell Systems) or in M199 medium supplemented with 20% FBS, 3 ng/mL recombinant human fibroblast growth factor-basic (FGF-b; Millipore, Temecula, CA), 5 U/mL heparin, penicillin (100 U/mL), and streptomycin (100 μg/mL) in a humidified atmosphere of 5% CO_2_ at 37 °C. Leukemia HL-60 cells were purchased from the Korean cell line bank (Seoul, Korea) and maintained in RPMI 1640 supplemented with 10% FBS, penicillin (100 U/mL), and streptomycin (100 μg/mL).

### Animal TNF-α injection

Male 7-week-old C57BL/6 mice were obtained from Daehan Biolink, Inc. (Eumsung, Chungbuk, Korea) and housed in clear plastic cages under specific pathogen-free conditions with a 12 h/12 h (light-dark cycle) and free access to standard irradiated chow (Purina Mills, Seoul, Korea). Mice were randomly divided into three groups with 3~4 individuals per group, and allowed to acclimate to their cages for at least 7 days before experiments. Mice were intravenously injected with saline or ML324 (0.2 mg/kg) for 4 h before TNF-α treatment, and sacrificed 24 h after intravenous injection with either saline or TNF-α (9 μg/kg). Dankook University Animal Care and Use Committee granted approval (1D: 13-033) for all experimental procedures involving animals. The methods were carried out in accordance with the approved guidelines.

### Transfection

Cells were plated in 6-well tissue culture dishes at a density of 2 × 10^5^ cells/well in complete medium and grown until they were approximately 90% confluent. Cells were transfected with 5 μg MSCVhygro-F-G9a plasmid DNA using Lipofectamine 2000 (Invitrogen, Carlsbad, CA) following the manufacturer’s instructions. After incubation at 37 °C for 6 h, cells were rinsed and supplied with fresh M199 complete medium.

For siRNA transfection, cells were plated in 6-well tissue culture dishes in complete medium and grown until they were approximately 60% confluent; they were then transfected with 100 nM siRNA using Lipofectamine RNAiMAX (Invitrogen) following the manufacturer’s instructions. siRNA oligonucleotides targeting ICAM1 and KDM4B and scrambled siRNA are listed in Table 1.

### Quantitative RT-PCR (qRT-PCR)

Total RNA was isolated from HBMVECs following the manufacturer’s instructions (NucleoSpin RNA; Macherey-Nagel, Düren, Germany). Next, 1 μg RNA was converted to cDNA using GoScript™ reverse transcriptase (Promega, Madison, WI), 2 mM MgCl_2_, 0.5 mM dNTPs, 20 U recombinant RNasin^®^ ribonuclease inhibitor and 0.5 μg oligo(dT)_15_. Reaction conditions were as follows: 25 °C for 5 min, 42 °C for 60 min, and 70 °C for 15 min. qRT-PCR was performed using 0.2−2 μL cDNA in a 20 μL reaction mixture, containing Fast SYBR^®^ Green Master Mix (Applied Biosystems, Foster City, CA) and primers. Reaction conditions were as follows: 95 °C for 10 min, followed by 40 cycles of 95 °C for 20 s, 60 °C for 15 s, and 72 °C for 20 s. Fluorescence was measured and analyzed with a 7500 Fast Real-time PCR system (Applied Biosystems). Relative gene expression was calculated using 7500 Fast software (Applied Biosystems). All PCR primer pairs are listed in Table 2.

### Western blot analysis

Cells were washed with ice-cold Dulbecco’s phosphate-buffered saline (DPBS; Hyclone, Logan, Utah), and then lysed for 30 min on ice using RIPA buffer containing 20 mM Tris-HCl pH 7.4, 150 mM NaCl, 1 mM ethylene diamine tetraacetic acid (EDTA), 1 mM ethylene glycol tetraacetic acid, 1% Triton X-100, protease inhibitor cocktail tablets (Roche, Penzberg, Germany), protein phosphatase inhibitor cocktail (Roche), and 1 mM PMSF. Protein concentration was determined using a BCA assay kit (Pierce, Rockford, IL). Equal amounts of protein (20 μg) were dissolved in sample buffer containing 50 mM Tris-HCl pH 6.8, 2% sodium dodecyl sulfate (SDS), 100 mM dithiothreitol, 0.01% bromophenol blue, and 10% glycerol and separated by 8% SDS-polyacrylamide gel electrophoresis (PAGE) under reducing conditions. This was followed by transfer onto polyvinylidene difluoride membranes. Blots were probed with appropriate antibodies (1:1,000): anti-ICAM1, anti-β-actin, anti-G9a, anti-KDM4B, or anti-VCAM1. Anti-rabbit and anti-mouse horseradish peroxidase-conjugated IgG (1:5,000) were used as secondary antibodies; the blots were subsequently developed using enhanced chemiluminescence (ECL) reagents (Advansta, Menlo Park, CA).

### Chromatin immunoprecipitation (ChIP) assay and semi-quantitative PCR

A ChIP assay kit (Upstate, Lake placid, NY) was used following the manufacturer’s instructions and as previously described^21^. Briefly, TNF-α-treated and non-treated HBMVECs were cross-linked in 2 mM disuccinimidyl glutarate and 1% formaldehyde, neutralized with 0.1 M glycine, and then suspended in SDS lysis buffer (1% SDS, 10 mM EDTA, and 50 mM Tris-HCl, pH 8.1). The chromatin solution was sonicated, pre-cleared, and immunoprecipitated with 0.2 μg/mL of desired antibodies (anti-H3K27me3, anti-H3K9me2, anti-H3K9me3, anti-H3K9ace, and anti-H3K14ace; Millipore) and A-agarose/Salmon Sperm DNA beads. Mock immunoprecipitations with anti-IgG served as controls. Input (total chromatin extract), mock and ChIP samples, were recovered and then used for semi-quantitative PCR analysis. PCR amplification was conducted in a total volume of 20 μL containing 0.1 units of TaKaRa Ex Taq HS polymerase (TaKaRa Bio INC, Shiga, Japan), 2 mM MgCl_2_, 0.2 mM dNTP, each primer (200 nM), 2 μL of DNA sample, and primers (listed in Table 2) recognizing the *Icam1* promoter region. Reaction conditions were as follows: 95 °C for 5 min, followed by 30 cycles at 95 °C for 30 s, 60 °C for 30 s, 72 °C for 30 s, and 72 °C for 5 min. The amplified products were separated on a 1.5% agarose gel in TAE buffer (40 mM Tris-acetate, pH 8.0, 1 mM EDTA). Bands were visualized using 1 μg/mL ethidium bromide and UV light.

### Adhesion assay

Confluent HBMVEC monolayers in 12-well plates were treated with 10 ng/mL TNF-α or 10 μM BIX01264 for 24 h, and then incubated for 0.5 h in the presence or absence of neutralizing antibodies (2 μg anti-ICAM1 and 2 μg anti-VCAM1). Leukocytes (HL-60; 10^5^) in 0.5 mL RPMI 1640 were added and incubated for 1 h to allow cell attachment. Non-adherent HL-60 leukocytes were removed and HBMVECs were washed three times with 1× DPBS to remove loosely attached cells. Attached HL-60 cells were detected under an inverted microscope at 400× magnification.

### Leukocyte transmigration assay

Leukocyte transmigration was performed using a Leukocyte transmigration assay kit (Cell Biolabs, Inc., San Diego, CA). Briefly, HBMVECs were seeded on a collagen type 1-coated 3-μm-pore-size polycarbonate membrane inserts in 24 trans-well plates and cultured in M199 growth media containing 20% FBS, 3 ng/mL bFGF and 5 U/mL heparin. Upon confluency (100%), HBMVECs were treated with BIX01294 (10 μM) or TNF-α (10 or 50 ng/mL) for 24 h, and exposed to neutralizing antibodies (anti-ICAM1 and anti-VCAM1) for 0.5 h. Then, HMBVECs were supplement with the RPMI1640 growth media containing 10^5^ HL-60 cells labeled with LeukoTracker^TM^, and this insert was transferred to a new 24 trans-well plate filled with 500 μL RPMI growth media. Leukocytes migrated across membrane of insert were collected in 24 trans-well for 24 h and 400 μL of the 500 μL medium containing migrating cells was placed in a clean well that contains 150 μL of 4× lysis buffer (Cell Biolabs, Inc.) and incubated at room temperature with shaking for 5 min. The mixture (200 μL) was transferred to a 96-well plate for fluorescence measurement at 480/520 nm.

### Tissue processing and double immunofluorescence staining

Anesthetized mice were perfused with 10 mM PBS (pH 7.4), and the brains were removed rapidly. Tissue was post-fixed with PBS containing 4% paraformaldehyde at 4 °C for 18 h, and transferred to 30% sucrose in PBS overnight. Brain tissue was frozen and serially sectioned (20 μm) using a sliding microtome (HM 450; Thermo Scientific, Walldorf, Germany). Sections were permeabilized with 0.03% triton X-100 and blocked with 5% normal goat serum for 2 h at room temperature. Samples were incubated with anti-ICAM1 mouse monoclonal and anti-collagen Type IV rabbit polyclonal (Fitzgerald, MA) antibodies at 4 °C overnight. After washing three times with 1× PBS, sections were incubated with Alexa Fluor^®^ 594 goat anti-mouse IgG and Alexa Fluor^®^ 488 goat-anti-rabbit IgG secondary antibodies at room temperature for 2 h, and were mounted using mounting medium. Localization of ICAM1 and collagen type IV in the brain was assessed using ZEN 2009 software on a Zeiss LSM confocal microscope (Carl Zeiss) equipped with a 20× objective lens.

### Neutrophil preparation and fluorescence staining

Neutrophils from mouse bone marrow were isolated as reported previously^48^. Briefly, bone marrow cells were flushed from mice femurs and tibias and briefly resuspended in 0.2% NaCl and 1.6% NaCl solutions, respectively, to remove red blood cells. Cells were loaded over Histopaque 1119 and 1077 and then centrifuged at 2,000 rpm for 30 min at 25 °C. The neutrophil pellet was washed with RPMI 1640 supplemented with 10% FBS and 1% P/S and incubated with 5 μM CellTracker Green (5-chloromethylfluorescein diacetate) at 37 °C for 10 min. Neutrophils (5 × 10^6^) in 200 μL PBS were injected into the lateral tail veins of mice.

### Quantitative analysis of fluorescent cells by confocal microscopy

Images were acquired from mouse brain sections using confocal microscopy (Leica, Germany) equipped with a 40× objective lens. Six 30-μm sections were taken every sixth section from each mouse brain starting from the anterior hippocampus. Fluorescent cells were counted by moving through the entire z-axis using Imaris software (Bitplane).

### Statistical analysis

Results are expressed as mean ± SEM. Data were analyzed by one-way ANOVA followed by *post hoc* Student-Newman-Keuls test; differences were considered significant for p < 0.05. A student’s *t-*test was applied for the analysis of significant differences between two groups. Calculations were performed using SPSS software (version 18.0; SPSS Inc., Chicago, IL).

## Additional Information

**How to cite this article:** Choi, J.-Y. *et al*. KDM4B histone demethylase and G9a regulate expression of vascular adhesion proteins in cerebral microvessels. *Sci. Rep.* **7**, 45005; doi: 10.1038/srep45005 (2017).

**Publisher's note:** Springer Nature remains neutral with regard to jurisdictional claims in published maps and institutional affiliations.

## Supplementary Material

Supplementary Information

## Figures and Tables

**Table 1:** siRNA nucleotide sequence.

Genes	siRNA sequence	
	Forward	Reverse
ICAM1	GCUCAAGUGUCUAAAGGAU	AUCCUUUAGACACUUGAGC
KDM4B	GGCAUAAGAUGACCCUCAU	AUGAGGGUCAUCUUAUGCC
Scramble	UUCUCCGAACGUGUCACGU	ACGUGACACGUUCGGAGAA

**Table 2:** Primer sequence.

Genes	Primer sequence	
	Forward	Reverse
KDM4B	ACGAGTAGGGACTGTGTCCA	GAAGATGTCCCCACGCTTCA
ICAM1	TGTGACCAGCCCAAGTTGTT	AGTCCAGTACACGGTGAGGA
VCAM1	CCAGTTGAAGGATGCGGGAG	ATGACCCCTTCATGTTGGCT
GAPDH	CCATGGAGAAGGCTGGGG	GGTCATGAGTCCTTCCACGA
ICAM1_ChIP 1	CTCTGGATGGCCAGTGACTC	CCGCCTAAGGCTTTCCTGTT
ICAM1_ChIP 2	GATCCAAGCTAGCTGCCTCA	CGTCCTCTCTCTACACCCGA
ICAM1_ChIP 3	GACCGTGATTCAAGCTTAGCC	GCTGCAGTTATTTCCGGACTG
ICAM1_ChIP 4	GCTATAAAGGATCACGCGCC	CAATCCCCACCCCGACTCA
β-actin_ChIP	CCAACGCCAAAACTCTCCC	AGCCATAAAAGGCAACTTTCG

**Table 3:** List of antibodies for ChIP assay.

Antibodies	Host	Company	Catalog number
Anti-H3K9ace	Rabbit polyclonal	Millipore	06–942
Anti-H3K14ace	Rabbit polyclonal	Millipore	07–353
Anti-H3K9me2	Mouse monoclonal	Millipore	05–1249
Anti-H3K27me3	Rabbit polyclonal	Millipore	07–449
Anti-H3K9me3	Rabbit polyclonal	Millipore	07–442
Anti-G9a	Rabbit monoclonal	Cell signaling	3306
Anti-KDM4B	Rabbit monoclonal	Cell signaling	8639
