# Supplementary material for: KDM4B histone demethylase and G9a regulate expression of vascular adhesion proteins in cerebral microvessels
Source: Sci Rep. 2017 Mar 22;7:45005. doi: 10.1038/srep45005 (PMC5361151; doi:10.1038/srep45005)
Supplement: Supplementary Information [file srep45005-s1.pdf]

KDM4B histone demethylase and G9a regulate expression of vascular adhesion proteins in cerebral microvessels

Ji-Young Choi[1], Sang-Sun Yoon[1], Sang-Eun Kim[2], and Sangmee Ahn Jo[1,2,*]

[1]Department of Nanobiomedical Science & BK21 PLUS NBM Global Research Center for Regenerative Medicine, Dankook University, Cheonan 330-714, South Korea

[2]Department of Pharmacology, College of Pharmacy, Dankook University, Cheonan 330-714, South Korea

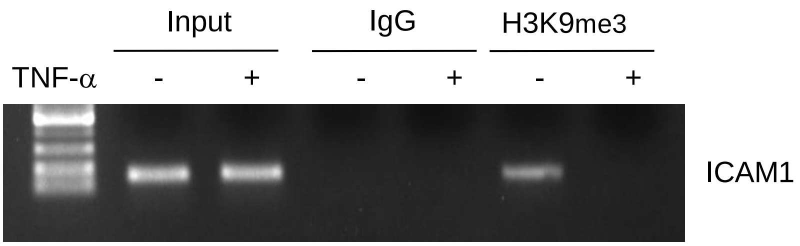

Supplementary Fig. S1. Effect of TNF-α on H3K9me3 level within the *Icam1* promoter in HBMVECs. A Chip assay was performed with antibody specific for H3K9me3. The DNA isolated after ChIP was used for a semi-quantitative PCR with ChIP 2 primer (listed in Table 2).

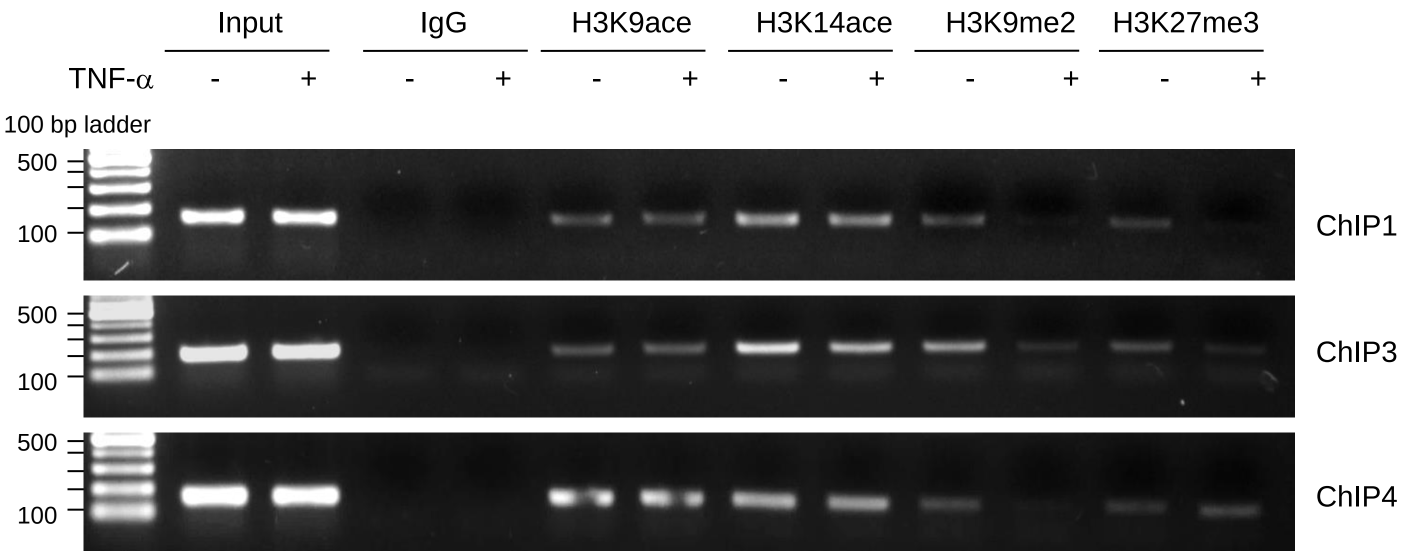

Supplementary Fig. S2. Effect of TNF-α on histone H3 lysine acetylation or methylation level within the *Icam1* promoter in HBMVECs. A Chip assay was performed with antibodies specific for H3K9ace, H3K14ace, H3K9me2, or H3K27me3. The DNA isolated after ChIP was used for a semi-quantitative PCR with ChIP1, 3, and 4 primers (listed in Table 2).

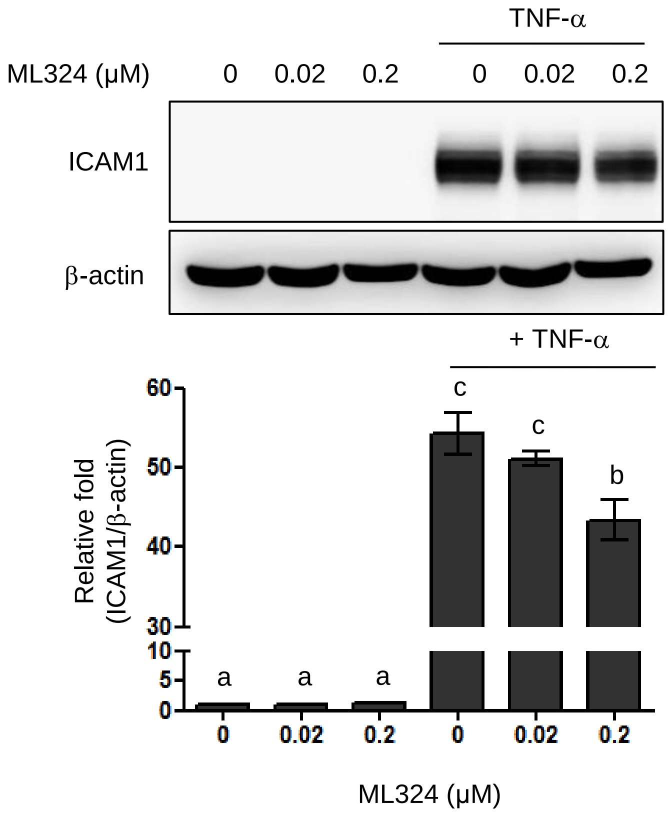

Supplementary Fig. S3. Effect of ML324 KDM4 inhibitors on ICAM1 expression. ICAM1 protein levels in HBMVECs pretreated with ML324 (0.02 and 0.2 μM) for 1 h prior to TNF-α treatment for 24 h. Quantification was performed using densitometry (Image J software). Results were normalized to β-actin. Bars represent mean ± SEM (n = 3). The different characters denote significant differences ($p < 0.05$) between the groups.

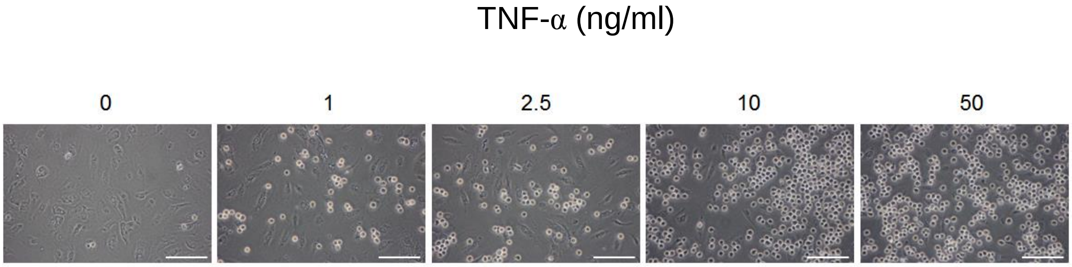

Supplementary Fig. S4. Effect of TNF-α on leukocyte adhesion. HBMVECs seeded in a 12-well plate were incubated with a various dose of TNF-α for 24 h. Attached HL-60 cells were observed under an inverted microscope with 400× magnification. Scale bars, 50 μm.

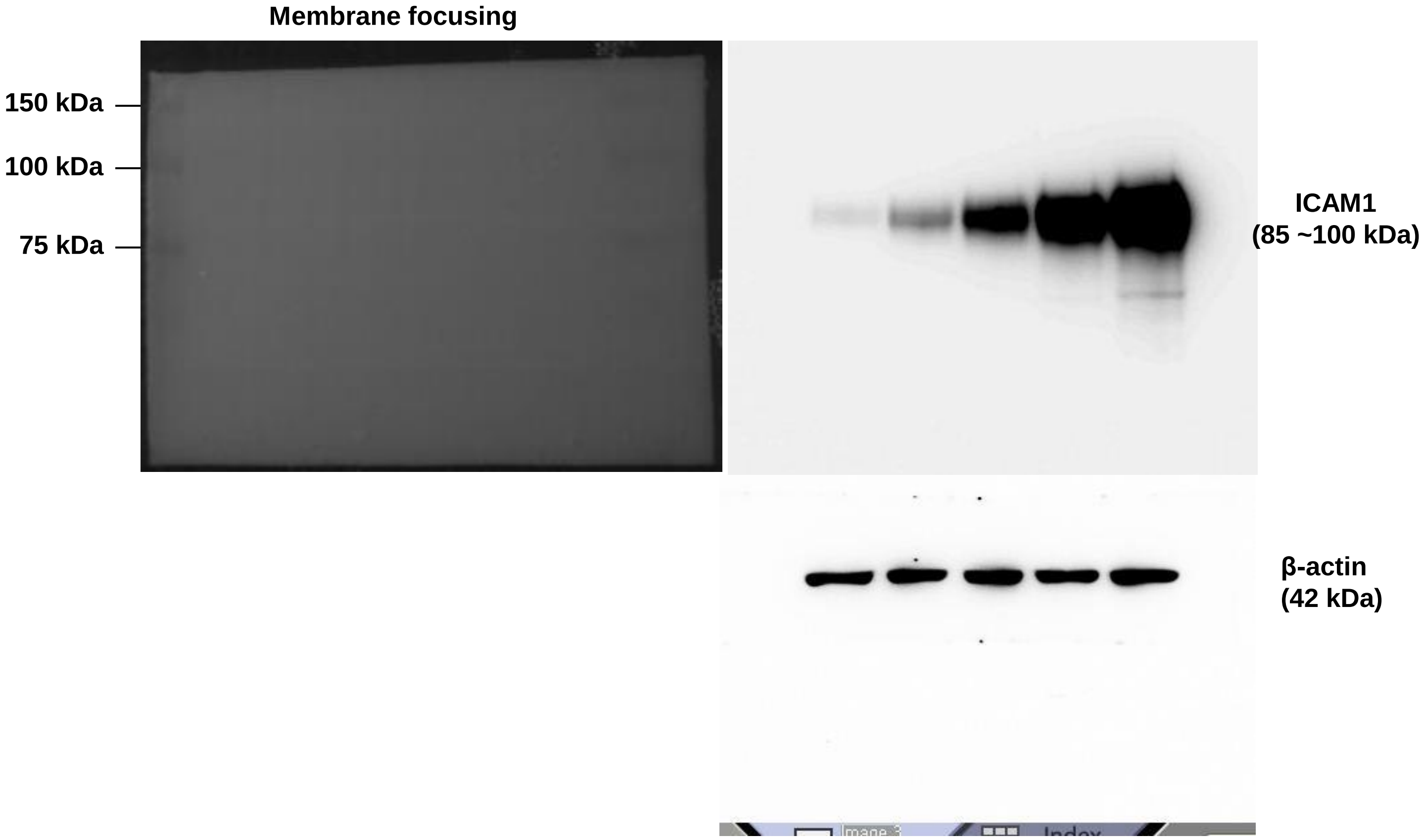

Supplementary Fig. S5. Uncropped image for Fig. 1b . 20 μg cell lysate was subjected to 8% SDS-PAGE. ICAM1 protein level was detected under ImageQuant LAS 4000 mini (GE Healthcare Life sciences). The protein standard marker (Bio-rad precision plus , # 161-0374) was loaded at the first lane on membrane to indicate the size of proteins. Blots incubated with anti-ICAM1 rabbit pAb (Satan-cruz, #sc-7891) were stripped to detect the β-actin protein level.

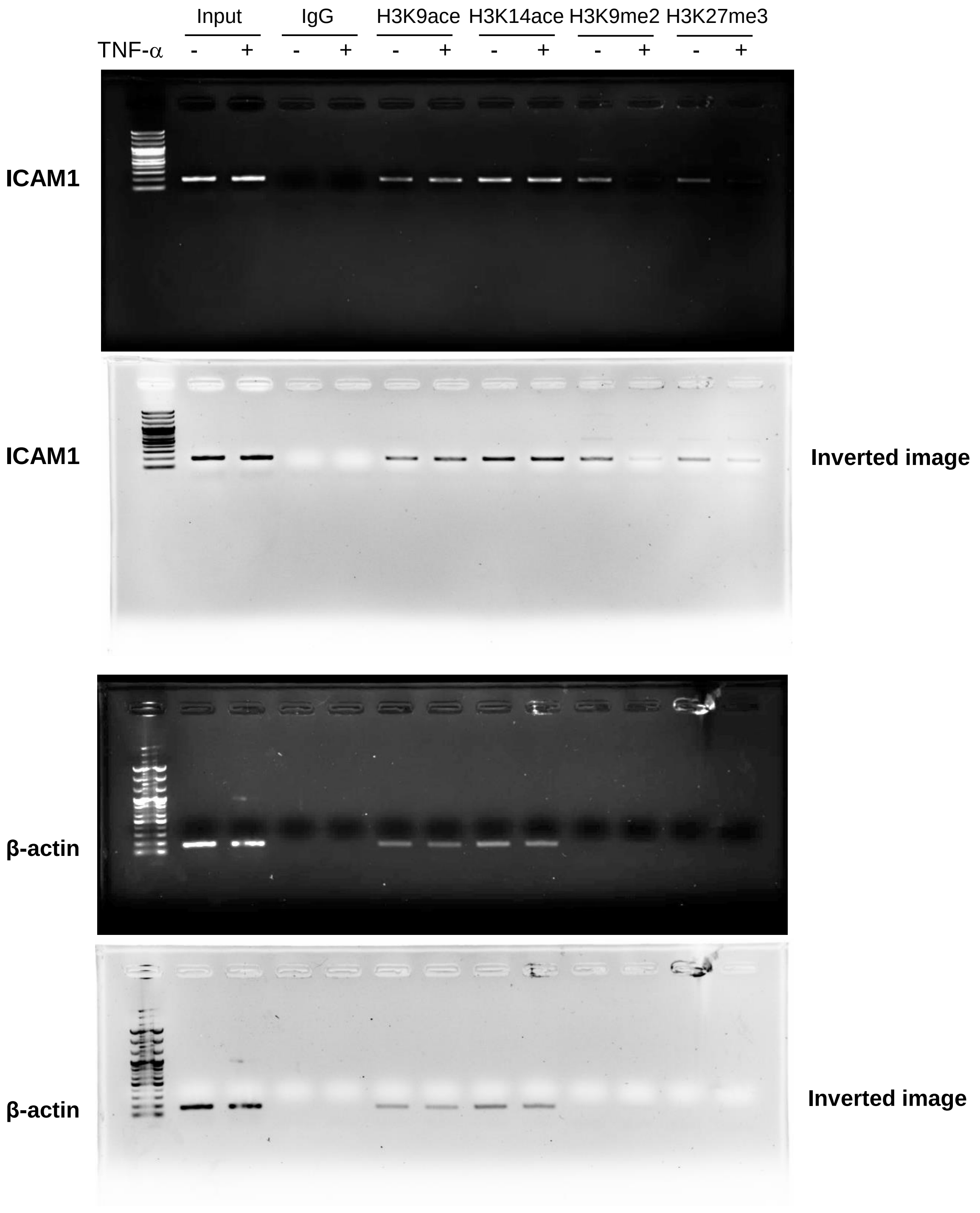

Supplementary Fig. S6. Uncropped image for Fig. 2b. 1% agarose gel stained with 1 μg/mL ethidium bromide was visualized using Molecular Imager GelDoc™ XR+ imaging system (Bio-Rad). Image was exported as a TIFF file using Image Lab 5.2.1 program (Bio-Rad; upper) and then inverted (bottom). 100 bp DNA ladder (Bio-Fact, Daejeon, South Korea; # SM342-100) was loaded at the first lane on the agarose gel.

(a)

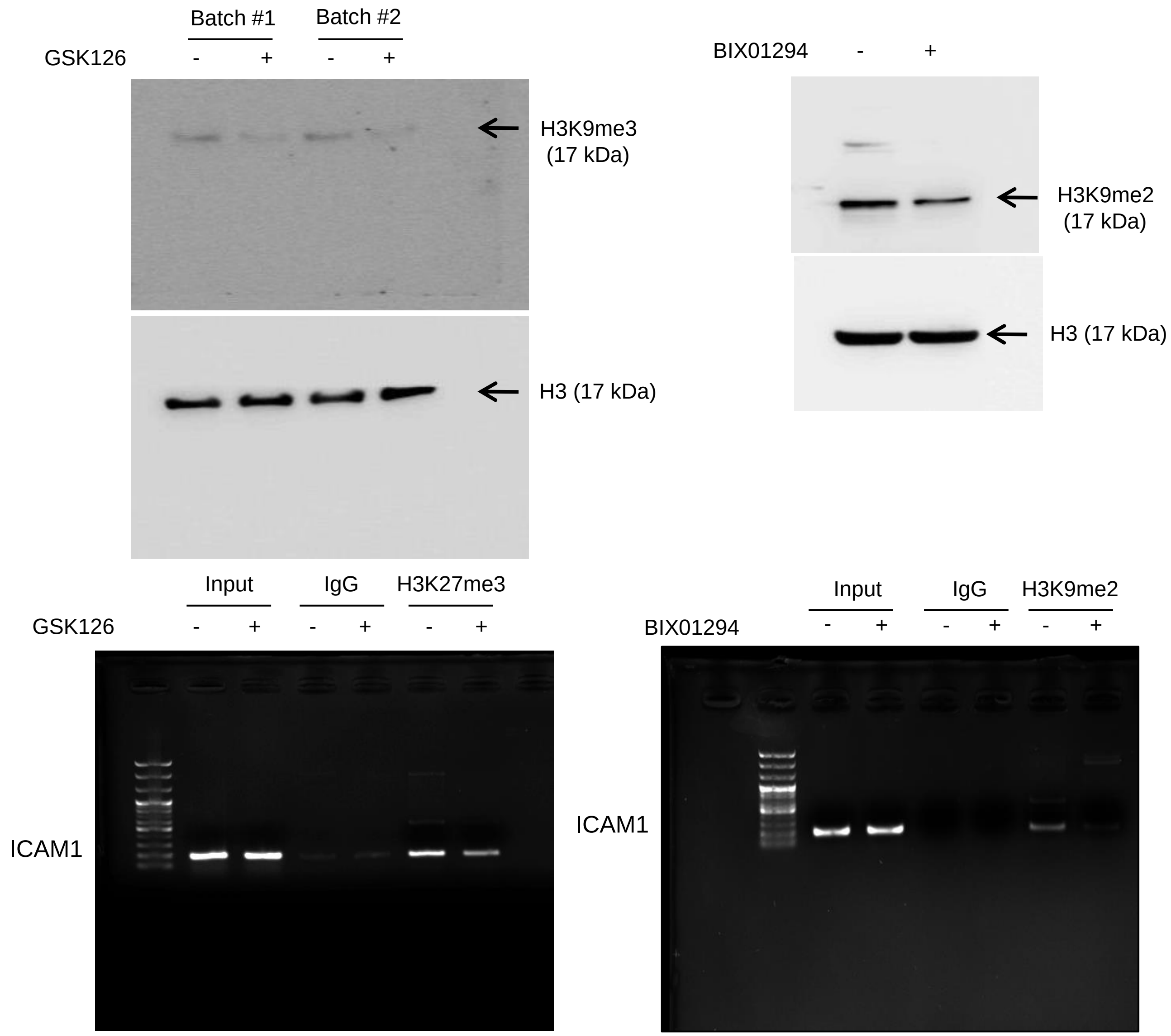

Supplementary Fig. S7. (a) Uncropped image for Fig. 3c . After detecting the H3K27me3 or H3K9me2 levels, the primary antibodies bound on the membrane were removed with Stripping buffer (# BSB-9000; Tech-innovation, Gangwon, South Korea), and then incubated with blocking solution containing anti-H3 antibody. The protein level of H3K27me3 and H3K9me was detected under ImageQuant LAS 4000 mini (GE Healthcare Life sciences). **(b) Uncropped image for Fig. 3d**

(a)

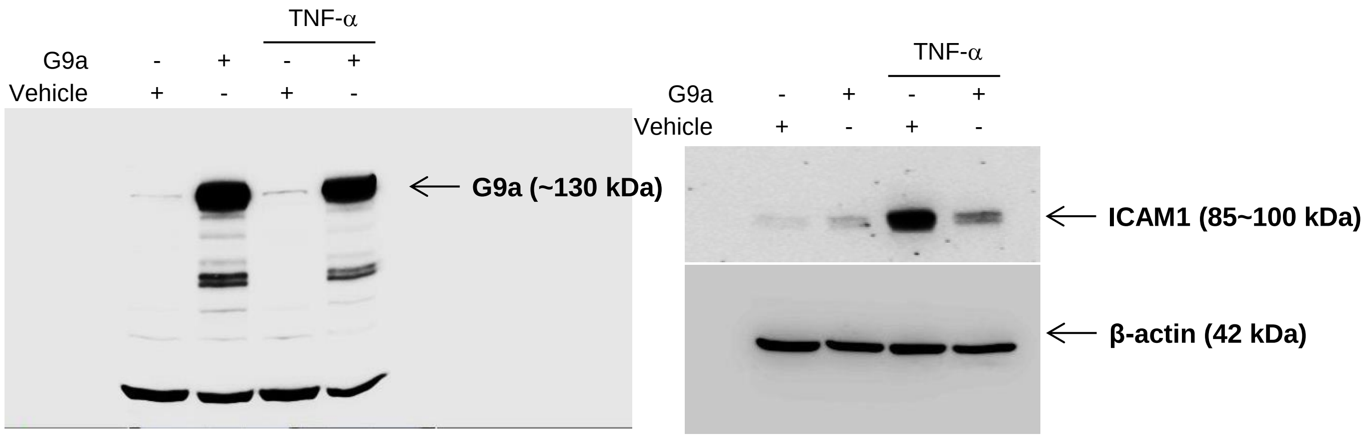

(b)

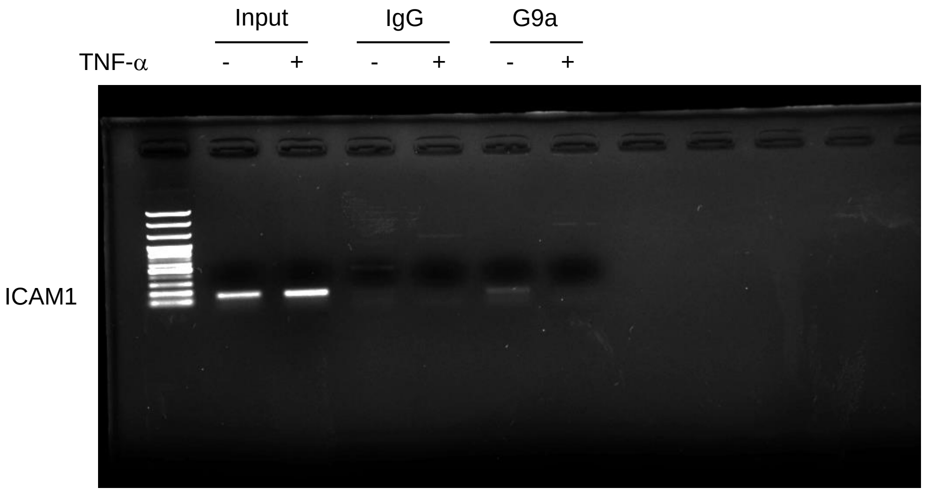

Supplementary Fig. S8. (a) Uncropped image for Fig. 4a. After detecting the G9a protein level, the blot was incubated in stripping buffer at RT for 30 min and divided into two pieces to detect ICAM1 (85 ~ 100 kDa) and β-actin (42 kDa). **(b) Uncropped image for Fig. 4b.**

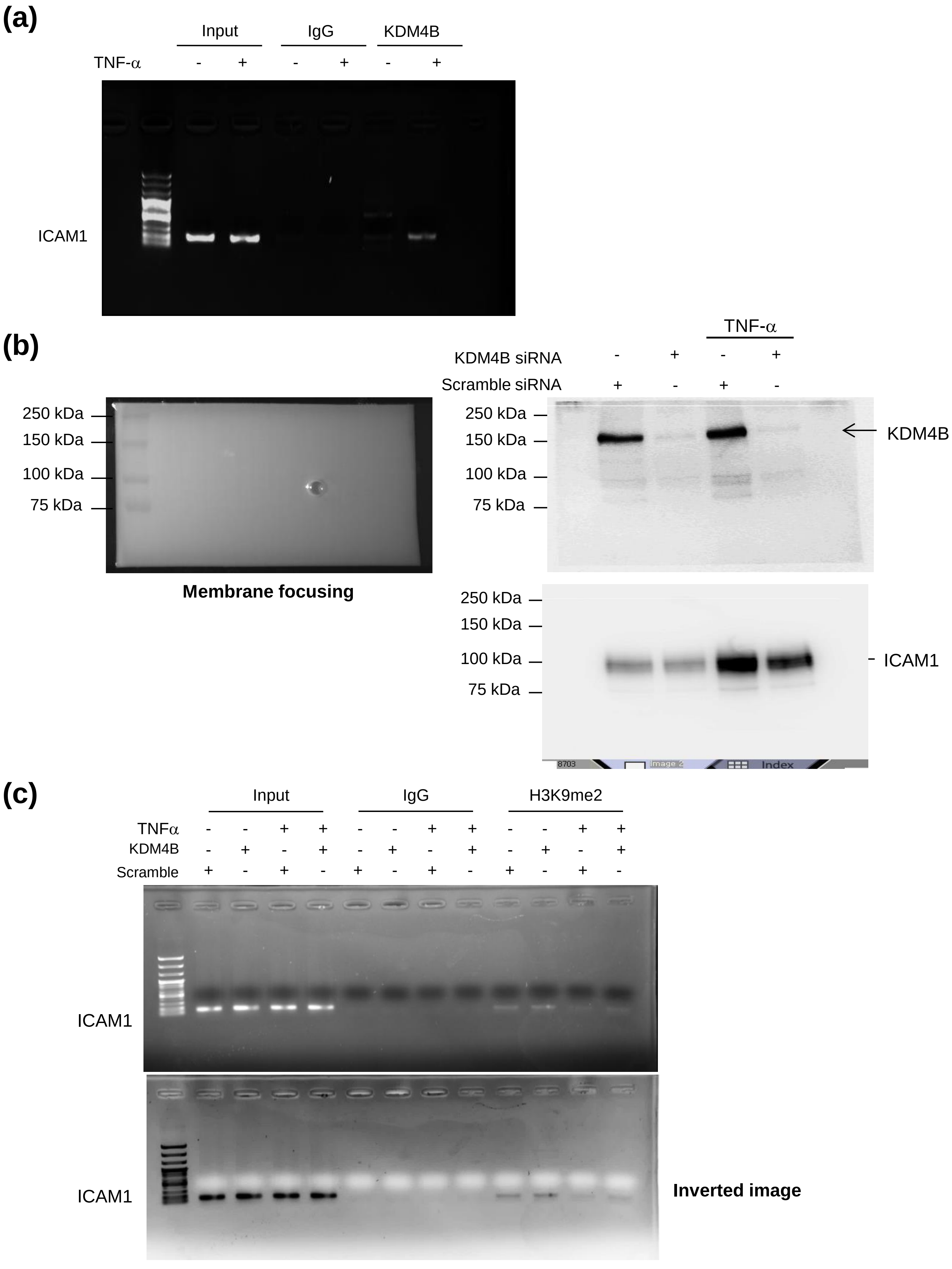
(a)
Input
IgG
KDM4B
TNF-α
- + - + - +
ICAM1
(b)
TNF-α
KDM4B siRNA
- + - +
Scramble siRNA
+ - + -
250 kDa
150 kDa
100 kDa
75 kDa
Membrane focusing
KDM4B
ICAM1
(c)
Input
IgG
H3K9me2
TNFα
KDM4B
Scramble
ICAM1
Inverted image

Supplementary Fig. S9. (a) Uncropped image for Fig. 5a. **(b) Uncropped image for Fig. 5d.** After detecting the KDM4B level, the blot was incubated in stripping buffer at RT and then processed to detect the ICAM1 protein level. The KDM4B and ICAM1 protein bands were detected under ImageQuant LAS 4000 mini (GE Healthcare Life sciences). **(c) Uncropped image for Fig. 5e.**

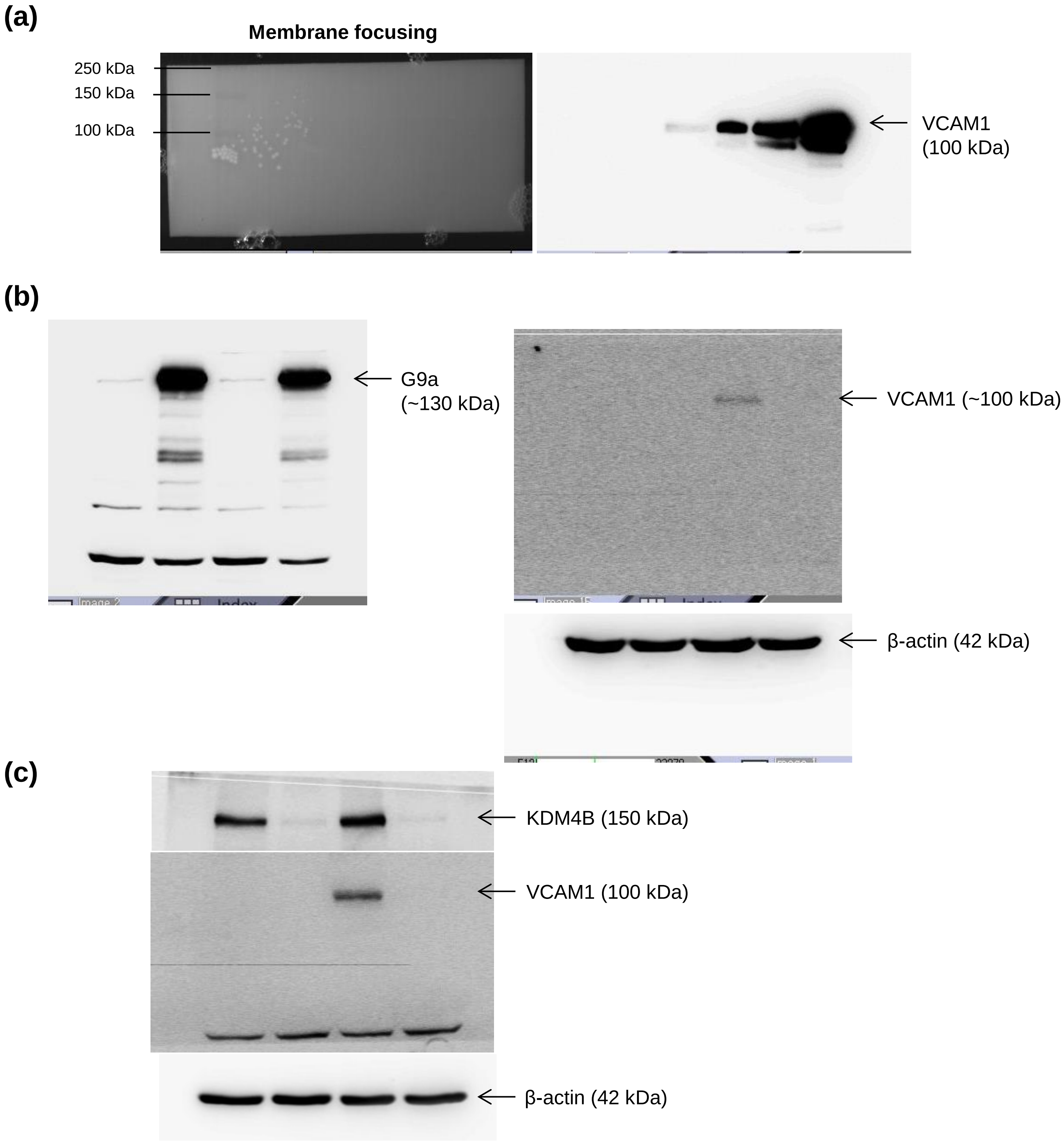

Supplementary Fig. S10. G9a and KDM4B regulates VCAM1 expression. **(a) Uncropped image for Fig. 6b.** VCAM1 band was detected at approximately 100 kDa. **(b) Uncropped image for Fig. 6c.** After detecting the G9a protein level, the blot was incubated in stripping buffer at RT for 30 min, and then divided into two pieces to detect the VCAM1 (100 kDa) and β-actin (42 kDa) protein levels. **(c) Uncropped image for Fig. 6d.** The blot was divided into three pieces to detect the KDM4B, VCAM1 and β-actin protein levels.
